# Assessing and Enhancing the Adequacy of Radiology Request Forms at a Tertiary Care Hospital in Sudan: A Clinical Audit

**DOI:** 10.7759/cureus.67187

**Published:** 2024-08-19

**Authors:** Abubakr Muhammed, Ahmed Mohamed, Mohand Tag Elsser Mohammed Albadwy, Ahmed Bhaeldin Ahmed Elbagir, Elshima Khaled Mohamed Khalfalla, Basil Mohammed Ahmed Hassan, Dina Hashim Wahid Eldin Osman, Ahmed Ibrahim Ishag Eltahir

**Affiliations:** 1 Department of General Surgery, Managil Teaching Hospital, Wad Madani, SDN; 2 Department of Orthopaedics, Gezira Centre for Orthopaedic Surgery and Traumatology, Wad Madani, SDN; 3 Department of Surgery, Atbara Police Hospital, Khartoum, SDN; 4 Public Health, Wad Madani Teaching Hospital, Wad Madani, SDN; 5 Department of Orthopaedics, Port Sudan Teaching Hospital, Khartoum, SDN

**Keywords:** quality improvement, royal college of radiology, standardization, request forms, radiology, clinical audit

## Abstract

Background: Clinical auditing is one method of improving the quality of patient care. Radiology request forms (RRFs) are crucial for the purpose of facilitating communication between the radiologist or radiographer and the referring clinician.

Methods: A two-cycle clinical audit was conducted at the Radiology Department of Atbara Police Hospital in Khartoum, Sudan. A total of 100 forms were collected, with 50 forms from each cycle. As an intervention, the existing blank paper format for radiology requests was replaced with a structured form based on the standards of the Royal College of Radiologists (RCR). This new form was distributed hospital-wide, and doctors were educated on its use through presentations and briefings. The collected forms were then compared against the RCR standards for completeness and accuracy. Data were entered into Microsoft Excel 2016 for analysis.

Results: The interventions led to notable improvements in several areas. Accuracy in recording the patient's address, phone number, and location increased from 0 (0%) in the first cycle to 50 (100%) in the second cycle, marking a 100% improvement. In addition, referencing the patient's age improved from 15 (30%) in the first cycle to 50 (100%) in the second cycle, indicating a 70% increase. The mean score for RRF documentation was 25.33% in the first cycle and significantly increased to 97.77% in the second cycle.

Conclusion: The audit shows significant improvement in RRFs post-intervention, highlighting the importance of standardization. However, deficiencies point to the need for orientation and follow-up training for physicians to ensure accurate form completion. Integrating quality assurance, including periodic audits and real-time feedback, can help sustain these gains. Collaboration between radiologists and referring physicians is also essential for ongoing improvement.

## Introduction

The process of evaluating current practices and making necessary modifications to improve the quality of patient care is known as a clinical audit, as defined by the National Institute for Health and Care Excellence (NICE) [1]. The drive of medical professionals to provide better services was a significant force behind its expansion in the UK, and clinical audits have been one of the most effective methods for achieving this goal. Regular clinical audits enhance the quality of a healthcare system [2].

Radiology request forms (RRFs) face several specific issues impacting patient care and the efficiency of radiological services. One major problem is incomplete information. Missing details, such as patient history or specific areas of concern, can hinder accurate image interpretation and lead to misdiagnoses or repeat imaging. In addition, inaccuracy in the provided information is another significant issue. Errors in patient identification or the requested imaging modality can result in incorrect studies, wasted resources, and delayed care. Illegibility, particularly in handwritten forms, exacerbates this problem, causing misinterpretations and delays.

Non-standardized formats across departments create confusion and inconsistency, and there is a lack of standardization among various institutions [3]. Without uniform structure, essential information might be omitted or misinterpreted, leading to inefficiencies and increased error risks. Timeliness is crucial; delays in form submission can postpone imaging studies, impacting diagnoses and treatments, especially in urgent cases.

Inadequate detail on forms, such as insufficient clinical history or unclear imaging indications, can hinder accurate interpretations and necessitate follow-ups, causing further delays. Data fragmentation, with information scattered across various systems, complicates the radiology process and increases the risk of oversight.

Clinicians complete the request form, and the radiographer, or in extraordinary circumstances, the radiologist, provides the test in accordance with the specified instructions. The radiologist interprets the request; therefore, the RRFs are indispensable communication instruments between the physician and the radiologist/radiographer, as they are used to refer patients for radiological examinations.

The Royal College of Radiologists (RCR) [4] mandates that requests be completed in a manner that is both precise and legible to prevent misunderstandings. The imaging specialist must be provided with sufficient clinical information to comprehend the specific diagnostic or clinical issues that the radiological examination is intended to address, and the justifications for the request must be explicitly stated.

The ordering physician and the treating physician are both accountable for promptly requesting the appropriate radiologic test and providing precise demographic information about the patient [5,6].

Previous research indicates that up to 20% of radiography examinations may be clinically ineffective due to incorrect or inappropriate requests. Consequently, it is imperative to provide adequate and pertinent information when requesting radiology examinations to enhance the effectiveness and utilization of radiological services [7]. The referring clinician or their authorized representative must complete the RRFs, which are both clinical and legal documents. The forms must specify the required procedure and its medical justification [4].

In general, Africa experiences a chronic shortage of radiology training programs, both at the diagnostic radiology (DR) residency level and the radiology specialty (RS) fellowship level [8]. Consequently, in Sudan, there are only a few radiologists in the entire city of Atbara (the subject of this research) and its surrounding areas. This is the first study on RRFs to be conducted in Sudan, as far as the authors are aware, which underscores the substantial knowledge gap. Furthermore, Sudanese hospitals have yet to implement the RCR's recommendations regarding RRFs as of the study's conclusion. The aim of this study was to assess and enhance the RRFs at Atbara Police Hospital in Sudan.

## Materials and methods

This prospective study involved an audit of 100 RRFs conducted at Atbara Police Hospital in Sudan, a prominent facility serving a diverse population, in May 2024.

Pre-intervention state

The initial phase of the audit involved a comprehensive assessment of the existing radiology request process. Fifty RRFs were randomly selected from the department's archives for examination. This initial analysis revealed significant deficiencies and inconsistencies in the radiology request process, emphasizing the necessity for improvement.

Root cause analysis

A root cause analysis was conducted to identify the underlying issues contributing to the deficiencies in the radiology request process. The primary issues identified included the use of an unstructured request form, lack of standardization across departments, and insufficient education and training for doctors on the proper completion of RRFs. In addition, during practice, we discovered that many pathologies were being misdiagnosed due to the clinical physicians writing nonspecific notes. As a result, the diagnostic outcomes were often ambiguous. This ambiguity frequently led to the need for retesting, which not only wasted valuable resources but also increased the burden on patients and the healthcare system.

Intervention

**Figure 1:**
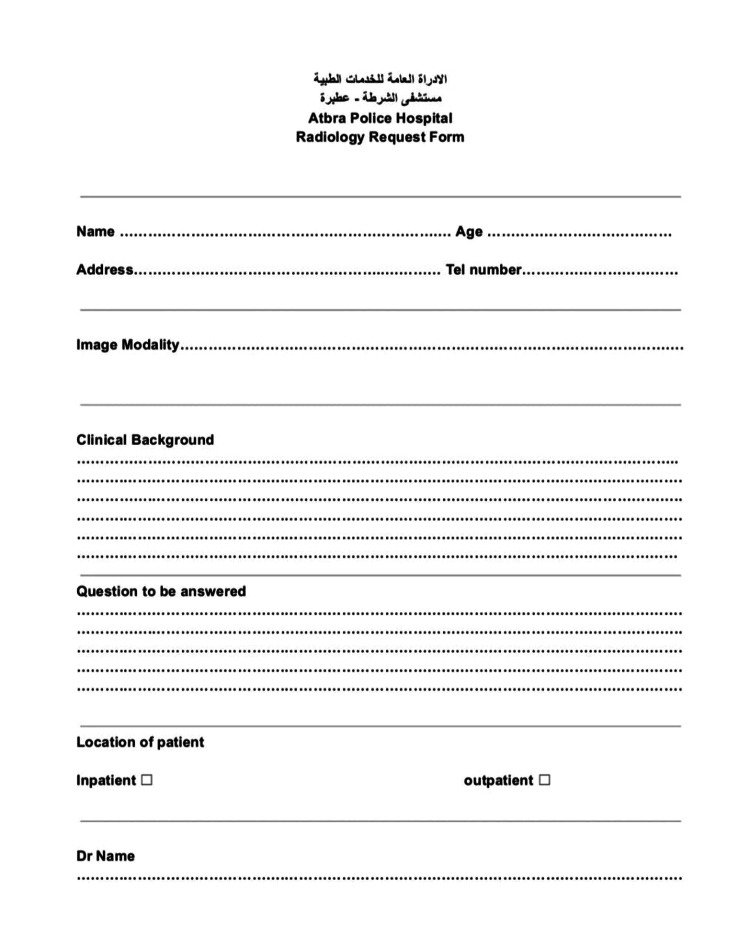
New radiological request form

To address the identified issues, a structured intervention was implemented. The intervention involved the following steps: (1) Development of a structured request form: The previous unstructured paper format for radiology requests was replaced with a new, structured request form that adhered to the standards set by the RCR (Figure 1). (2) Distribution of the new form: The new request form was distributed to various hospital departments to standardize the submission process. (3) Education and training: Doctors were educated on the importance and proper use of the new form through a combination of methods, including a PowerPoint presentation during departmental meetings, focused group discussions, individual briefings, and posters distributed throughout the hospital.

Post-intervention state

Following a two-week intervention period, the second cycle of the audit commenced. Fifty RRFs were randomly selected and analyzed to assess the impact of the intervention. The analysis aimed to evaluate improvements in the radiology request process and adherence to RCR guidelines. 

Data analysis

The collected data from both audit cycles were analyzed using Microsoft Excel® 2016 (Microsoft Corporation, USA). Descriptive statistics and graphical representations were employed to ensure a thorough assessment and clear presentation of the findings. The forms included requests for X-rays, ultrasounds, and echocardiograms in the first cycle, while CT scan forms were also included in the second cycle due to the acquisition of a new CT machine by the hospital.

Evaluation

Four authors independently evaluated the RRFs, comparing them against the RCR guidelines. In cases where discrepancies occurred between the evaluations, a third party was consulted to resolve conflicts and ensure the accuracy and reliability of the audit findings.

Ethical approval

This study was approved by the Institutional Ethics Committee (IEC) at Atbara Police Hospital under reference number ATA-11. All personal and clinical data were anonymized prior to analysis to ensure privacy and confidentiality.

## Results

A comprehensive comparison of the completeness of RRFs against established standards is provided in Table 1, which spans two distinct cycles. The degree of adherence to a 100% compliance standard in both the first and second cycles was determined by meticulously evaluating each criterion within the forms. The table illustrates the level of completeness and accuracy that was achieved before and after the implementation of the RCR standards, including patient identification, clinical history, and specific diagnostic requests.

The study examined a total of 100 RRFs, with 50 forms collected from the first cycle and an additional 50 from the second cycle. In the first cycle, the forms included requests for X-rays, ultrasounds, and echocardiograms. However, by the second cycle, CT scan forms were also included as the hospital had acquired a new CT machine, which was previously only available in private institutions.

**Figure 2:**
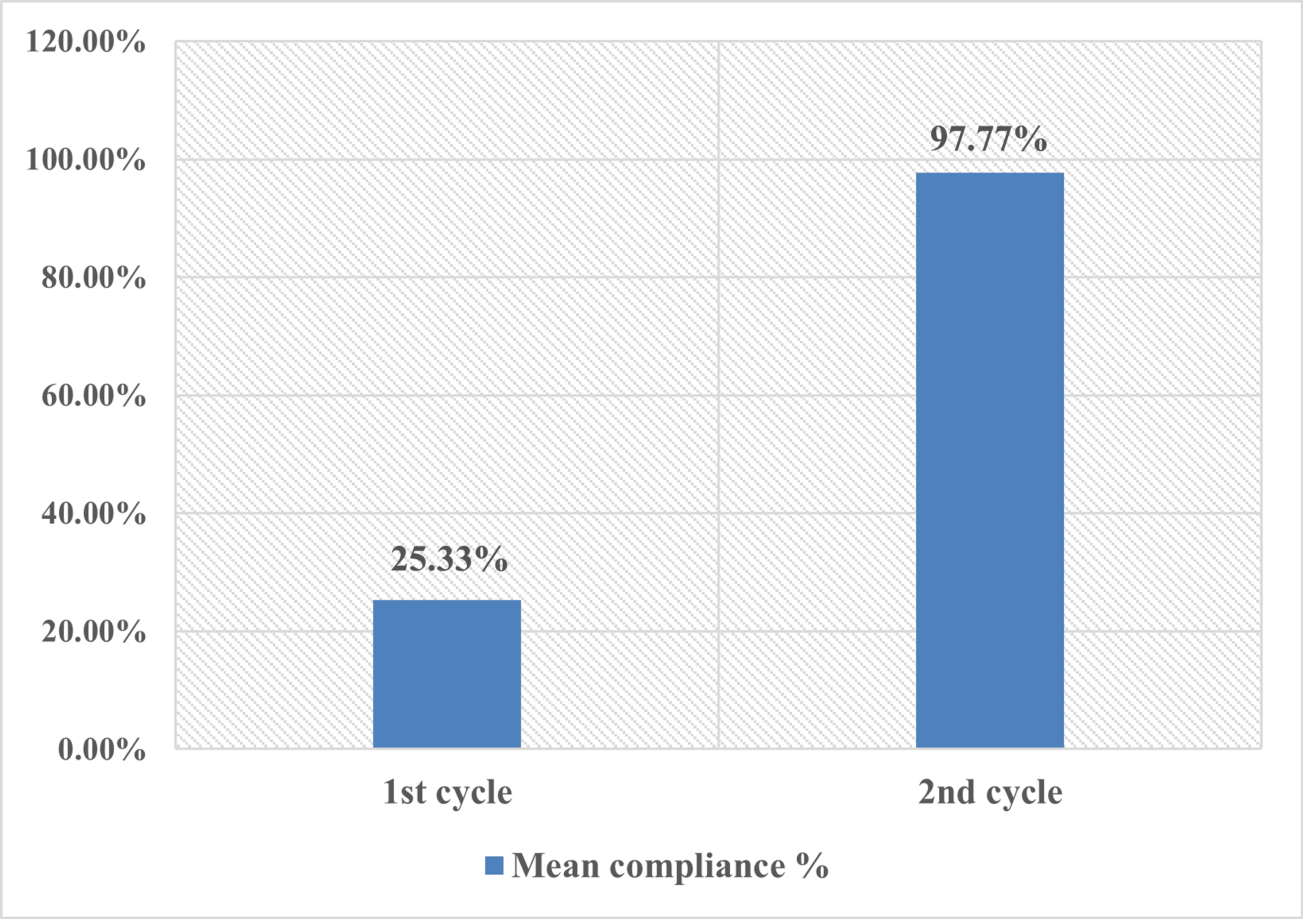
Compliance percentages in the first and second cycles

Initially, as illustrated in Figure 2, only 25.33% of the RRFs adhered to the RCR's recommendations during phase 1. Nevertheless, the RCR standards' introduction and implementation resulted in significant enhancements in compliance. This substantial improvement is demonstrated by the remarkable increase in the success rate of the RRFs, which increased to 97.77% during the second cycle. This enhancement emphasizes the efficacy of adhering to the RCR standards in enhancing the precision and quality of RRFs.

The interventions resulted in a substantial improvement in the majority of areas. For instance, the first cycle's accuracy in recording the patient's address, phone number, and location, as well as in addressing the question, increased significantly from 0 (0%) to 50 (100%) in the second cycle, a 100% improvement. Furthermore, the frequency of referencing the patient's age increased significantly from 15 (30%) in the first cycle to 50 (100%) in the second cycle, indicating a 70% improvement. Table 1 shows the evaluation results and percentage improvement for RRFs.

**Table 1:** Evaluation results and percentage improvement for radiology request forms

Criteria	First cycle N (%)	Second cycle N (%)	Improvement %
1. The clinical background	26 (52%)	50 (100%)	48
2. The question to be answered	0 (0%)	50 (100%)	100
3. The patient’s name	50 (100%)	50 (100%)	-
4. The patient’s age	15 (30%)	50 (100%)	70
5. The patient’s address	0 (0%)	50 (100%)	100
6. The patient’s number	0 (0%)	40 (80%)	80
7. Location of the patient	0 (0%)	50 (100%)	100
8. Name of the requesting practitioner	23 (46%)	50 (100%)	54
9. Question to be answered	0 (0%)	50 (100%)	100

## Discussion

The incomplete completion of radiography request forms is a global concern [9]. In general, patients receive the most effective care when all teams involved implement a multidisciplinary approach [4,7,9]. RRFs frequently function as the primary method of communication between clinicians and radiologists as a result of the limited opportunities for regular discussions on clinical cases and management [10]. Nevertheless, radiologists or radiographers may directly contact the managing clinician or inquire with the patient for additional information prior to the procedure.

The findings of this investigation suggest that none of the request forms were adequately completed during the initial cycle. This is consistent with the findings of Akinola et al. [11] and Depasquale and Crockford [9], who discovered that only 4% of request forms were completed in their entirety. In addition, the patient's ward or address was fully completed in 100% (n = 50) of the request forms in the second cycle of this study, in contrast to the findings of Depasquale and Crockford [9], who found that 77% of request forms included addresses, and Akinola et al. [11], who found that only 2.1% had addresses. The imaging department is able to submit reports for the majority of inpatients by including addresses, thereby reducing the waiting time.

The patient's name is the initial step in ensuring accurate patient identification [12]. The findings of this investigation indicated that the names of the patients were accurately entered on all forms. Agi et al. [13] observed that all the forms under examination had the patients' names accurately filled in a related study conducted in Nigeria.

In contrast to Agi et al. [13], who had 100% of the investigated stated questions answered, none of the request forms from the first cycle had stated questions that needed to be addressed. The patient was required to provide the radiologist with a clinical history. These omissions may result in an increase in patient dissatisfaction, transportation costs, and waiting times. It was noted that the question that required an answer was included in 100% (n = 50) of the request forms during the second cycle. The absence of clinical information may result in medical errors or a delay in initiating the appropriate treatment, both of which can have a significant adverse impact on the safety and outcome of patients in the future [14].

The referring doctor is responsible for collecting all diagnostic information that justifies the requested radiological examinations, as well as information about previous exposures, in accordance with the relevant articles of the Radiation Protection Regulations of the European Union Nations [15]. The clinical question to be answered and the clinical information provided is essential for the radiologist to determine whether to conduct the requested investigation, prevent the patient from receiving unnecessary radiation, and assist in making a final or differential diagnosis [9,11].

Our study demonstrated that none of the physicians who referred patients provided their contact information to enable the radiologist to engage in further communication. This finding is in direct opposition to the standards established by the RCR, which require referring practitioners to provide contact information [4]. Regardless of any perceived challenges, a multidisciplinary management team should prioritize effective communication between radiologists and referring clinicians.

There were numerous constraints associated with this investigation. Initially, the audit was conducted at a single location, which may restrict the generalizability of the results. Furthermore, the sample size was relatively small, further limiting the generalizability of these findings. It is also crucial to mention that the medical staff was aware that their RRFs were being monitored, which may have resulted in temporary adherence to the guidelines. Based on observations conducted within the radiology department of Atbara Hospital in Sudan, the conclusions drawn from this audit are specific to this context. Consequently, it is probable that these results cannot be extrapolated to other departments or institutions due to differences in workflow, patient volumes, and resource availability.

## Conclusions

The results of this two-cycle audit indicate that RRFs experienced substantial enhancements subsequent to intervention. The findings emphasize the importance of standardization in improving the quality of RRFs. However, the identified deficiencies highlight the need for orientation and follow-up training for physicians in referral teams to ensure they understand the importance of accurately completing these forms. This type of training is crucial to ensure that radiologists have access to all necessary information to accurately diagnose patients and provide the highest quality of care.

Furthermore, integrating a quality assurance mechanism into the workflow could help sustain the improvements achieved. This mechanism might include periodic audits and real-time feedback to ensure continued adherence to documentation standards. Encouraging collaboration between radiologists and referring physicians can also foster a better understanding of the information required and promote a culture of continuous improvement in documentation practices.
